# Interval Safety Layer Coupled With an Impulsive MPC for Artificial Pancreas to Handle Intrapatient Variability

**DOI:** 10.3389/fendo.2022.796521

**Published:** 2022-02-21

**Authors:** María F. Villa-Tamayo, Maira García-Jaramillo, Fabian León-Vargas, Pablo S. Rivadeneira

**Affiliations:** ^1^ Universidad Nacional de Colombia, Facultad de Minas, Grupo GITA, Medellin, Colombia; ^2^ Universidad EAN, Facultad de Ingeniería, Grupo ONTARE, Bogotá, Colombia; ^3^ Universidad Antonio Nariño, Facultad de ingeniería Mecánica, Electrónica y Biomédica (FIMEB), Grupo REM, Bogotá, Colombia

**Keywords:** artificial pancreas, insulin on board, interval model, model predictive control, safety layer, type 1 diabetes

## Abstract

The aim of control strategies for artificial pancreas systems is to calculate the insulin doses required by a subject with type 1 diabetes to regulate blood glucose levels by reducing hyperglycemia and avoiding the induction of hypoglycemia. Several control formulations developed for this end involve a safety constraint given by the insulin on board (IOB) estimation. This constraint has the purpose of reducing hypoglycemic episodes caused by insulin stacking. However, intrapatient variability constantly changes the patient’s response to insulin, and thus, an adaptive method is required to restrict the control action according to the current situation of the subject. In this work, the control action computed by an impulsive model predictive controller is modulated with a safety layer to satisfy an adaptive IOB constraint. This constraint is established with two main steps. First, upper and lower IOB bounds are generated with an interval model that accounts for parameter uncertainty, and thus, define the possible system responses. Second, the constraint is selected according to the current value of glycemia, an estimation of the plant-model mismatch, and their corresponding first and second time derivatives to anticipate the changes of both glucose levels and physiological variations. With this strategy satisfactory results were obtained in an adult cohort where random circadian variability and sensor noise were considered. A 92% time in normoglycemia was obtained, representing an increase of time in range compared to previous MPC strategies, and a reduction of time in hypoglycemia to 0% was achieved without dangerously increasing the time in hyperglycemia.

## 1 Introduction

Managing type 1 diabetes (T1D) has proven to be challenging. People with T1D need exogenous insulin to regulate their blood glucose (BG) levels. The therapy required involves a risk of severe hypoglycemia, with all its consequences, if the insulin dose is too high. For this reason, the therapeutic goal is to minimize the number of hypoglycemic episodes and maximize time in the healthy glycemic range, known as normoglycemia zone. Accordingly, automated insulin delivery, also known as the artificial pancreas (AP), has emerged as the best solution to automatically modulate insulin and remove the threat posed by hypoglycemia (1, 2).

The control strategies more extended in the literature for AP systems are based on model predictive control (MPC) (1), proportional-integral-derivative (PID) (3), and fuzzy logic (4). There are several studies evaluating AP performance using different control strategies that have shown efficiency in clinical and free-living-condition trials (5–7). However, AP systems with dedicated safety schemes and/or adaptive laws are preferred over traditional control systems to reduce the risk of hypoglycemia in both fully closed-loop or hybrid developments (8–10).

Different adaptive control formulations based on MPC have been developed either to update the parameters of glycemia-insulin-carbohydrate models or to directly tune the control parameters. Strategies as those developed in (11, 12) and summarized in the review made by (13) consist of identifying the prediction model at every fixed period or when a condition is triggered like the detection of variations in glucose behavior. On the other hand, in (9, 14, 15), adaptive MPC strategies were formulated to change the penalization matrices of the MPC according to the current situation of the glycemia. Other works like (16–18) update other parameters of the strategy as the basal insulin, insulin-carbohydrate ratio, or the set-point, based on historical data and risk indexes to improve the controller performance.

In addition, the amount of active insulin that remains in the body, also known as insulin on board (IOB), has been used in several closed-loop safety schemes to prevent the insulin overdose, especially for the postprandial period (19–21). Different IOB models have been proposed to be part of open- and closed-loop controls with hypoglycemic prevention strategies (8, 22). In any case, an accurate value of the patient’s duration of insulin action (DIA) is required for a good performance.

Insulin pumps used in AP developments work with DIA values ranged from 2 to 8 hours in order to adjust their prevention strategies, as well as for the bolus calculators in hybrid systems (22, 23). A dynamic IOB constraint with estimated insulin action decay curves was first incorporated into the MPC problem by (20) and introduced in the safety module of the strategy proposed in (24). This idea was extended in (25, 26) where adaptive IOB rules were defined as a function of BG levels and the insulin delivery history was also considered to set the IOB boundary. Also (27), proposed an IOB decaying curve to set the input reference in the MPC cost function. The direct estimation of the IOB from a minimal physiological model was used in (28) where a control law was derived to avoid hypoglycemia. Additionally, a sliding-mode safety layer that runs outside the main control strategy was developed in (8) where the control action is modulated according to a surface computed as the difference between the estimation of the IOB and an IOB boundary. This strategy has been included in different works. In (29), the safety layer was coupled with a PID. In (30), the layer was coupled with a Linear Quadratic Gaussian (LQG) controller, and the IOB boundary was set as a piece-wise constant limit that changes according to a meal classification. In (6), the authors proposed a dynamic IOB boundary that depended on a factor of the open-loop IOB profile. In (10), it was shown that a single IOB boundary does not fit every situation of BG fluctuations as physiological variations in the subject change insulin requirements. Thus, a preliminary safety layer with adaptive IOB boundary was introduced.

Indeed, it has been shown that the range of insulin decay curves encompasses a variety of sources of uncertainty affecting the patient’s DIA, modifying the pharmacokinetics of the rapid-acting insulin analogues used in AP systems, whose value is usually between 3 and 5 hours (31). Common sources of uncertainty affecting how long it takes to absorb insulin, and thus an accurate estimate of IOB, are the patient’s insulin sensitivity, exercise, or heat.

Studies have revealed that insulin absorption can vary 10-30% in an individual and 20-50% between individuals (22). This can lead to overdoses of insulin when a shorter DIA value than actual is used in safety schemes based on the IOB, triggering episodes of hypoglycemia since the algorithm assumes there is less IOB than there actually is. In addition, it has been proved that selecting an inappropriate DIA setting is a common deficiency among clinicians and insulin pump users due to widespread misunderstanding of its concept (23).

Uncertainty associated to DIA setting, and therefore to the IOB estimation, has been addressed previously for AP designs through a method known as modal interval analysis (MIA) (32), where it is possible to consider a parametric uncertainty present in a dynamic model to be mathematically rewritten as an interval model (33). MIA allows to obtain a feasible simulation of the interval model providing an envelope of all the possible responses according to the percentage of parametric uncertainty established, avoiding under- and overestimation.

So far, only one AP design proposal implementing a safety scheme with MIA has been made (32) which was coupled with a PID-type controller. Although an IOB interval estimate was used, it was not implemented to generate the IOB boundaries required in the safety layer, and instead, the boundaries were chosen to be constant and to act like additional tuning parameters. Thus, there is no adaptation in this approach. In fact, when there are physiological variations towards hyperglycemia, the upper IOB limit (which is constant) could not be enough to allow the required insulin doses, and then prolonged hyperglycemia occurs.

This paper addresses the limitation found in previous works (referred to as the lack of consideration of the uncertainty in the IOB boundary selection) to obtain a control strategy that includes an adaptive safety module. To that end, the impulsive offset-free zone MPC (iZMPC) developed in (34) was used as the main controller, and concepts from the safety layer (6), and the uncertainty captured with the interval model in (32) were considered to build a new scheme. Now, the main features of the overall strategy are: (i) the safety layer uses the IOB interval model to generate upper and lower IOB boundaries that act as envelopes of the possible IOB trajectories; (ii) an auxiliary signal is generated to estimate the plant-model mismatch mainly given by physiological variations; (iii) with the information of the current glycemia, the estimated mismatch, and their corresponding trends and second derivatives, adaptive rules are developed to select the IOB boundary that is more appropriate for the current situation of the subject; (iv) with the selected IOB boundary at each time step, a gain is computed to modulate the control action of the MPC when required, and thus, to switch between aggressive and conservative control actions. To the best of the authors’ knowledge, this is the first time that the estimation of the plant-model mismatch, its derivatives, and the derivatives of the glycemia are used to adapt the safety layer.

A demanding assessment scenario with circadian variations in insulin sensitivity, the hepatic autoregulation, and endogenous glucose production, as well as comparisons with previous MPC designs, were considered in order to highlight the main improvements achieved. With the strategy in here formulated, a 92.7% of time in normoglycemia was obtained in comparison to the 83.8% resulting with the baseline iZMPC strategy and the 82.8% obtained with the preliminary strategy with safety layer developed in eViLeoGarRiv2021. In addition, the time in hypoglycemia was reduced to 0% in comparison to the 2.1% and 1.5% obtained with the previous strategies.

## 2 Methods

### 2.1 Glucose-Insulin-Carbohydrates System Dynamics

#### 2.1.1 Impulsive System Dynamics for Glucose Control

The impulsive scheme for glucose control is selected to emulate the natural treatment of T1D. This because insulin doses are administered as small, spaced pulses rather than a continuous or a discrete input. Here, the impulsive discretization of a minimal physiological model based on five compartments to represent glucose dynamics, insulin absorption and action, and meal absorption dynamics is used. It is a linear model that satisfy global structural identifiability (35). The state-space representation of the model is given by


(1) $$\begin{array}{l} x(k+1)=Ax(k)+B_{u}u(k)+B_{r}r(k)+E,\\ y(k)=Cx(k), \end{array}$$

where matrices are related with their continuous counterpart considering a fixed sampling time *T* as $A=e^{A_{c}T}$ , $B_{u}=e^{A_{c}T}B_{uc}$ , $B_{r}=\int_{0}^{T}e^{A_{c}s}dsB_{rc}$ , $E=e^{A_{c}T}E_{c}$ , with:


(2) $$A_{c}=\left[\begin{array}{ccccc} -p_{0} & -p_{1} & 0 & p_{2} & 0\\ 0 & -1/p_{4} & 1/p_{4} & 0 & 0\\ 0 & 0 & -1/p_{4} & 0 & 0\\ 0 & 0 & 0 & -1/p_{5} & 1/p_{5}\\ 0 & 0 & 0 & 0 & -1/p_{5} \end{array}\right],B_{uc}=[\begin{array}{ccccc} 0 & 0 & 1 & 0 & 0 \end{array}]',\;B_{rc}={\left[\begin{array}{ccccc} 0 & 0 & 0 & 0 & \frac{1}{p_{5}} \end{array}\right]}^{\prime },$$

*E_c_* = [*p*_3_ 0 0 0 0]*'*, and *C* = [1 0 0 0 0]. All model variables and parameters have physiological interpretation as seen in **Table 1**. The model parameters have been identified for the 10-adult virtual subjects available in the commercial version of the UVA/Padova simulator (36). The identification process is detailed in (37), and the resulting parameters, personalized for each subject, can be seen in **Table 2**. In addition, an analysis of the properties of the impulse model can be found in the **Appendix**.

**Table 1:** Description of variables and parameters of the model.

Variable	Description	Units
*x*_1_	Glycemia	mg/dl
*x*_2,_ *x*_3_	Insulin in the blood and subcutaneous space compartments, respectively	U
*x*_4,_ *x*_5_	Delivery rates of carbohydrates in the stomach and gut, respectively	g/min
*u*	Exogenous insulin	U/min
*r*	Carbohydrates intake	g/min
**Parameter**	**Description**	**Units**
*p*_0_	Hepatic autoregulation.	1/min
*p*_1_	Insulin sensitivity rate.	mg/dl/U/min
*p*_2_	Carbohydrate bioavailability.	mg/dl/g
*p*_3_	Endogenous glucose production at zero-insulin level.	mg/dl/min
*p*_4_	Time-to-maximum of effective insulin concentration.	min
*p*_5_	Time-to-maximum appearance rate of glucose.	min

**Table 2:** Model parameters identified from the 10-adult cohort of the UVA/Padova simulator.

Subject	*p*_0_	*p*_1_	*p*_2_	*p*_3_	*p*_4_	*p*_5_
Adult 1	0.0034	0.7896	2.3080	1.3270	56.001	21.840
Adult 2	0.0063	1.3544	2.4100	2.0110	40.004	14.624
Adult 3	0.0010	0.4841	1.7370	0.7570	52.202	21.516
Adult 4	0.0027	0.9806	2.9610	1.2520	59.502	25.429
Adult 5	0.0022	1.0654	3.8710	1.0360	46.782	28.638
Adult 6	0.0081	0.7140	4.1730	2.1810	52.503	23.511
Adult 7	0.0018	1.6722	4.3790	1.9010	47.505	22.023
Adult 8	0.0028	0.7683	4.3700	1.0570	50.007	26.854
Adult 9	0.0058	1.7445	4.4590	2.0730	50.505	24.461
Adult 10	0.0032	0.6849	2.5100	1.0740	50.503	23.317

#### 2.1.2 Insulin-On-Board Model

An IOB model allows to estimate how much insulin is still to act in the body, which depends on the pharmacokinetics of the insulin analogue used and the patient’s glucose-insulin dynamics. To perform an IOB estimation, it is necessary to know the corresponding duration of insulin action (DIA) of the patient. In practice, there is no agreed-on standard for DIA. Many patients and clinicians enter inappropriately short DIA times into insulin pumps even though practical recommendations have been suggested for a proper choice (23).

In this work, the IOB model is obtained from subsystem (*x*_2,_ *x*_3_) of model (1) using the IOB mathematical definition: 


(3) $$IOB(k)=x_{2}(k)+x_{3}(k),$$

representing the amount of insulin in the subcutaneous and plasma compartments from previous boluses. This model is consistent with IOB models where the corresponding DIA time is implemented through *K_DIA_*, a parameter that has been characterized to emulate typical DIA times (8). In model (3), *K_DIA_* ≡1/*p*_4_, and its value has been previously identified for each virtual subject considered here, see **Table 2**.

The Euler discrete time approximation of *x*_2_ and *x*_3_ of model (3), is given by


(4) $$\begin{array}{c} x_{3}(k+1)=Tu(k)-TK_{DIA}x_{3}(k)+x_{3}(k),\\ x_{2}(k+1)=TK_{DIA}(x_{3}(k)-x_{2}(k))+x_{2}(k), \end{array}$$

where *u(k)* is the insulin dose. This representation is used to obtain the IOB interval model considered in this work.

#### 2.1.3 Insulin-On-Board Interval Model

One of the main challenges in AP control is the intra-patient variability that has to be taken into account. In different works as (38, 39), a variability of 30% has been observed and used to evaluate control performance. This uncertainty is represented here by an interval model in which the parameters, inputs, and/or initial states take interval values (33).

**Figure 1:**
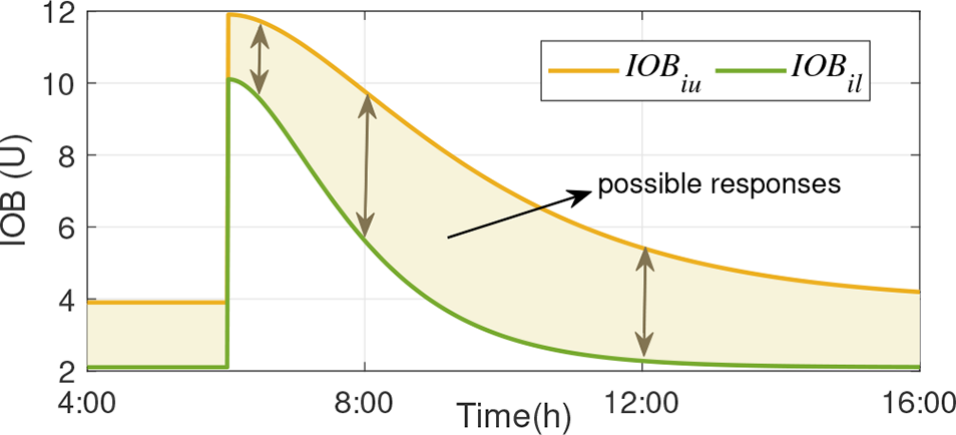
Envelopes obtained from the interval IOB model: Upper and lower bounds that define the possible system responses (shaded area).

The simulation of a model with particular real values for the parameters, starting from any initial state, yields trajectories of the output variables across time. When the quantities involved in the simulation take values inside the intervals of variation, the set of trajectories determines a plane band bounded by an envelope, as depicted in **Figure 1**. At each time step of the simulation, the envelope, i.e., the possible maximum and minimum values of the variable, must be determined. This is a range computation problem. The function whose range must be determined is defined by the interval model of the system, and the parameter space is determined by the interval values of the parameters, the input, and the initial state. The simulation of an interval model provides intervals (ranges) that can be estimates of the envelopes. These envelopes can be obtained by numerical integration, qualitative reasoning, fuzzy logic, etc. A way to compute these estimates is by interval arithmetic. However, the exact range of a function is not always computable. Therefore, the results are often very over bounded, and if tighter results are needed, high computational efforts are required.

To consider uncertainty in the IOB model (3), a modal interval analysis (MIA) is applied here to get an interval representation. MIA provides a strong theoretical background for dealing with problems involving uncertainty and logical quantifiers. In contrast to techniques such as Monte Carlo Simulation (MCS), MIA computational time is independent of the number of uncertain parameter. Thus, worst-case analysis can be performed efficiently, which is extremely important in the context of diabetes. A comparison between MCS and MIA was presented in (33). For further information on MIA, the reader can consult the appendix presented in (40).

MIA allows to obtain the whole range of possible responses of the model with uncertainty but avoiding, or minimizing under some conditions, the overestimation of interval computations. Overestimation is one of the main problems that arises from the existence of multiple instances of the same variable (multi-incident variable) with uncertainty. In model (4), uncertainty is considered through the patient’s *K_DIA_* parameter, implementing it as $K_{DIA_{i}}=[0.7K_{DIA},1.3K_{DIA}]$ .

To achieve an optimal computation, the IOB model (4) was initially analyzed to try to obtain its optimal form (the expression is rewritten in such a way that the exact range is obtained) to avoid multi-incident interval variables. This was possible for *x*_3_ but not for *x*_2_ of model (4), and therefore the theorem of coercion to optimality from MIA was also applied. To do this, the monotonic behaviour of *x*_2_ was studied with respect to the uncertain variable *K_DIA_*, leading to create a new interval state to obtain an optimal rational computation for the IOB model (3) as follows:


(5) x3i(k+1)=x3i(k)(1−ΔtKDIAi)+Δtu(k),x23i(k+1)=x23i(k)(1−ΔtKDIAi)+Δtu(k)+ΔtDual(KDIAi)x3i(k)x2i(k+1)=x23i(k+1)−Dual(x3i(k+1)),IOBi(k)=x3i(k)+x2i(k),

where the time step Δ*t*, used for the interval model simulation, is defined so that constraint $\Delta t<1/x_{23_{i}}$ holds according to the theorem of coercion to optimality from MIA. $x_{3_{i}}$ and $x_{2_{i}}$ are interval representations of *x*_3_ and *x*_2_ in (1), respectively, and $x_{23_{i}}$ is the new interval state created to avoid under- and overestimation of the model. Note that, $x_{3_{i}}$ , $x_{2_{i}}$ , $x_{23_{i}}$ , and *IOBi* are 2-dimension vectors containing lower and upper values. The dual operator is defined as *Dual* ([a_1_, a_2_]) = [a_2_, a_1_], according to MIA.

The output of this interval model corresponds to an upper (*IOB_iu_*) and a lower (*IOB_il_*) bound, that delimit an IOB envelope, see **Figure 1**. The envelope formed by these bounds is determined by the set of IOB trajectories involved in the simulation from all the possible responses taking values inside the interval of variation of the parameters with uncertainty (33). It is noteworthy that the output of the model is not simply the nominal response of the IOB in (3)by a factor of ±30%, in which case the envelope would be the same width all the time. On the contrary, as seen in Figure fig:IOBint, the width of the envelope, representing the range of possible instances, is also dynamic.

### 2.2 Control Strategy With Interval Safety Layer

#### 2.2.1 Impulsive Model Predictive Control

In T1D treatment, the control objective is to maintain the system’s state within a safety zone *X^Tar^* selected inside the desired range 70 mg/dl ≤ BG ≤ 140mg/dl. Bearing the above in mind, the MPC formulation to be used here is the impulsive offset-free zone MPC (denoted as iZMPC for simplicity). This formulation was developed in (34) and used in T1D context in (15, 41). The optimization problem solved by the iZMPC at every time step *k* is


(6) $$\begin{array}{l} \underset{u,x_{a},u_{a}}{\min}V_{N}(x,X_{s}^{Tar},U_{s}^{Tar};u,x_{a},u_{a})\\ s.t.x(0)=\hat{x}(k),d(0)=\hat{d}(k),\\ x(j+1)=Ax(j)+B_{u}u(j)+B_{r}r(j)+B_{d}d(j)+E,\\ d(j+1)=d(j),\\ u(j)\in U,x(j)\in X,\\ x(H_{p})=x_{a},\\ y_{a}=Cx_{a}+C_{d}d(j),\\ x_{a}=Ax_{a}+B_{u}u_{a}+B_{d}d+E. \end{array}$$

with cost function stated as


(7) $$V(x,X_{s}^{Tar},U_{s}^{Tar};u,u_{a},x_{a})=\sum_{j=0}^{H_{p}-1}∥x(j)-x_{a}{∥}_{Q}^{2}+\sum_{j=0}^{H_{u}-1}∥u(j)-u_{a}{∥}_{R}^{2}+P\left(dist_{X_{s}^{Tar}}(x_{a})+dist_{U_{s}^{Tar}}(u_{a})\right).$$

From this iZMPC problem, it is to remark the following features:

1. The decision variables are ***u***, which represents the control policy, and (*x_a_*,*u_a_*) ϵ (*X_s_*, *U_s_*), which are artificial equilibrium variables to be reached in the prediction horizon *H_p_*, where the sets *X_s_*, *U_s_* denote the equilibrium sets for system (1). The control horizon is denoted by *H_u._*
2. The last term of the cost function ensures that the final point reached in the prediction remains within the target zone, where $dist_{X_{s}^{Tar}}(x_{a})$ denotes the distance of *x_a_* to set $X_{s}^{Tar}$ , and $X_{s}^{Tar}$ , $U_{s}^{Tar}$ are generalized equilibrium sets for the target (42).
3. The state is augmented with a disturbance *d*(*k* + 1) = *d*(*k*) to handle a constant plant-model mismatch and avoid offset error. Thus, the model is extended as *x*(*k* + *1*)*=Ax*(*k*)*+B_u_ u*(*k*)*+B_r_r*(*k*)*+B_d_d*(*k*) + *E*, *y*(*k*) = *Cx*(*k*) + *C_d_d*(*k*), with $B_{d}=e^{A_{c}T}B_{dc}$ and *B_dc_* selected such that the augmented model be observable. In (34) it was shown that *B_dc_* = [100 0 0 0 0]*'* satisfies this condition. Then, the augmented state is estimated with a state estimator to capture both the system state $\hat{x}(k)$ and the mismatch $\hat{d}(k)$ and it is used to initialize the optimization problem. In this work, the Kalman Filter is implemented.
4. In the MPC problem (6), the prediction model and equilibrium constraints are corrected to consider the disturbance effect, i.e., to consider *d*.
5. In this work, the control strategy has been designed as hybrid, in which meal announcement is provided to the controller by using the term *B_r_r*(·) in the prediction model. In addition, sensing and actuation delays are not considered in the design of the control strategy, thus, the strategy should be able to compensate for these additional challenges despite the lack of information about it.
6. To solve the optimization problem at each time step, the quadprog solver of Matlab was used with the interior-point-convex algorithm and with tolerances TolPGG= 1×10^-5^, Tolcon=1×10^-4^, TolX= 1×10^-4^, and Tolfun=1*×*10^-4^ (see (43) for more details on the selection of the solver for quadratic programming in artificial pancreas context).

#### 2.2.2 Interval Safety Layer

**Figure 2:**
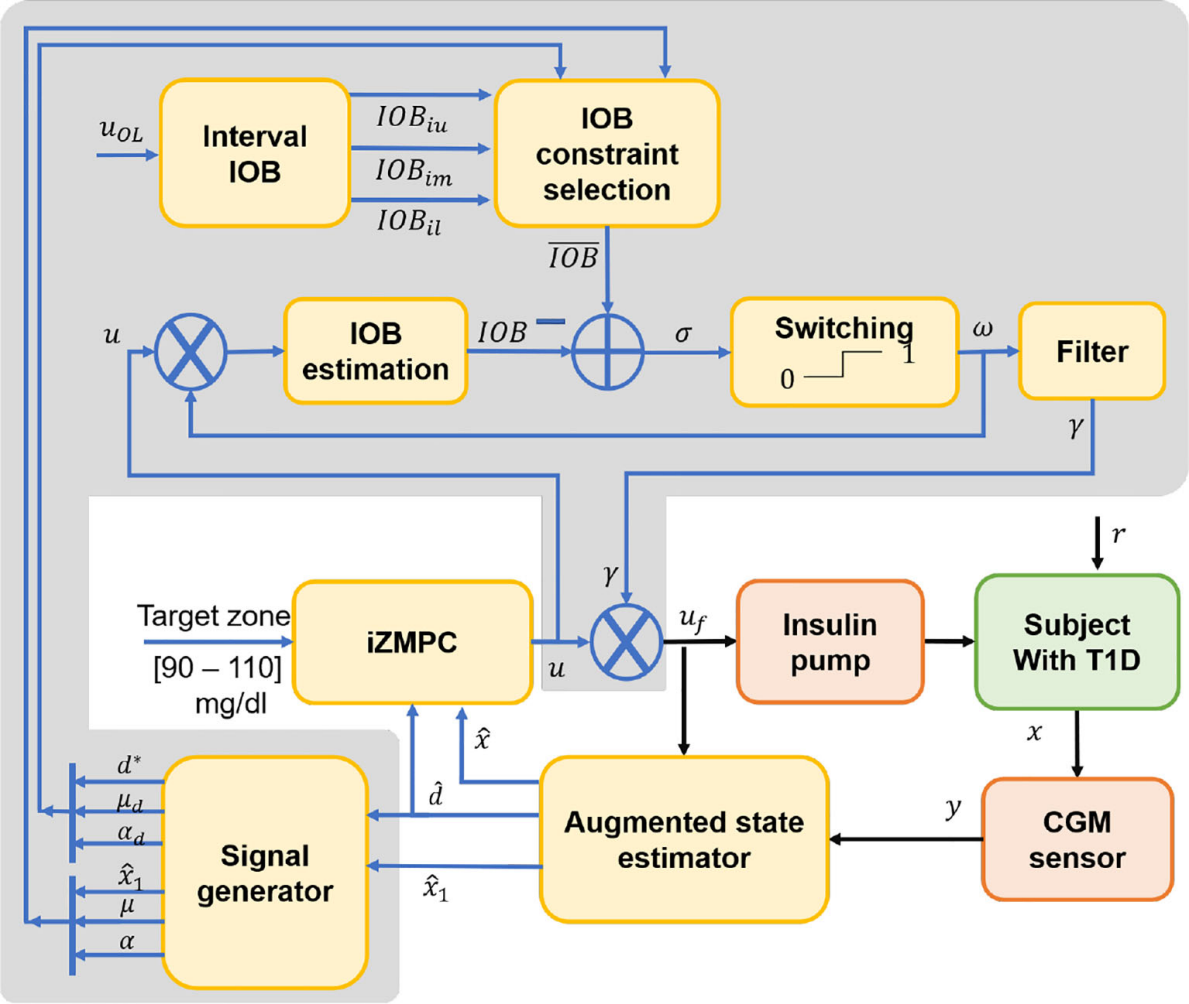
Block diagram of the iZMPC strategy with the interval layer coupling.

The main idea of the interval safety layer is to modulate the control action computed by the iZMPC to avoid hypoglycemia events. To this end, a gain *γ* ϵ [0 1] is calculated, such that the final command to the insulin pump *u_f_* (*k*)=*γ*(*k*)*u*(*k*) satisfies an imposed constraint on the IOB. As shown in **Figure 2**, the layer consists of 6 blocks, from which the IOB is estimated and compared to a boundary selected to be safe in the current situation of the system.

First, the interval IOB is performed to obtain the upper (*IOB_iu_*) and lower bound (*IOB_il_*). In addition, a middle band (*IOB_im_*) is obtained as the middle point between *IOB_iu_* and *IOB_il_*. These values are obtained with model (4) and the input *u*_OL_ corresponding to the open-loop insulin dose that should be administered to the subject, i.e., *u*_OL_ = *u_basal_* for fasting periods, and *u*_OL_ = *CHO/CR* when a meal is announced. *CHO* corresponds to the carbohydrates ingested, and *CR* is the insulin-to-CHO ratio (35).

Next, from the three values calculated (*IOB_iu,_ IOB_il_* and *IOB_im_*), the IOB constraint $\bar{IOB}$ has to be selected. From previous works as (15), it was shown that the sign of the estimated plant-model mismatch provides information about the direction of the physiological variations occurring in the patient. For instance, a negative $\hat{d}$ suggests variations inducing hypoglycemia, and a positive $\hat{d}$ suggests variations towards hyperglycemia. To prevent measurement noise from affecting the information provided by *d* and to smooth the effect of food intake ton *d*, a moving horizon filter is used as $d^{*}(k)=\Sigma_{k-N_{p}}^{k}d(k)/N_{p}$ , where *Np* is the postprandial window usually between 1-2 hours (15). Here, the idea is to select the IOB constraint of the safety layer by using the information of the plant-model mismatch, but in addition, by including the rate of change of d* (or its first time derivative, *μ_d_*), and its acceleration (or second time derivative, *α_d_*), as well as information of the BG value, and its first *μ* and second time derivatives *α*. All these values are obtained from the signal generator block that receives information from the augmented state estimator and produces the derivatives by numerically computation using backward differentiation. The information obtained from these six signals helps to anticipate the variations and glucose behavior, and thus, to take action accordingly. The following rules have been posed to commute between aggressive and conservative control actions:

##### 2.2.2.1 Case 1

The BG value is in the zone of imminent hyperglycemia, and it is detected that its value and rate of change are increasing (140 ≤ *BG* ≤ 180, *μ* ≥ 0, and *α* ≥ 0):

- If in addition the sign of the estimated mismatch has an increasing tendency (*μ_d_* ≥ 0), then a risk of variations inducing hyperglycemia are considered too. Therefore, an aggressive response is established by allowing the delivery of the complete control action *u* computed by the iZMPC, i.e. the IOB constraint is released (u*_f_* = *u*).

- If the variations are not increasing, then a less aggressive action is posed, but still allowing high doses of insulin. In this case, the IOB constraint is set as $\bar{IOB}=IOB_{iu}$ .

##### 2.2.2.2 Case 2

The BG value is not in the zone of imminent hyperglycemia, thus, the control action changes according to the information provided by the estimated mismatch:

- If either *d ≤ –ϵ*, or *μ_d_* < 0 and *d* ≤ *ϵ*, then a risk of variations inducing hypoglycemia is detected, and thus, the lower band is established as the IOB constraint to obtain conservative control actions $\bar{(IOB}=IOB_{il})$ .
- If *d >–ϵ* and *μ_d_* ≥0 and *α_d_* ≥0, then a risk of variations towards hyperglycemia is detected, thus, aggressive actions are allowed by changing the IOB constraint to the upper band $\bar{IOB}=IOB_{iu}$ .
- In any other cases, in which it is detected that the variations are near to a change of direction, then softer control actions than rule 2.2 are allowed by using the middle band $\bar{IOB}=IOB_{im}$

**Figure 3:**
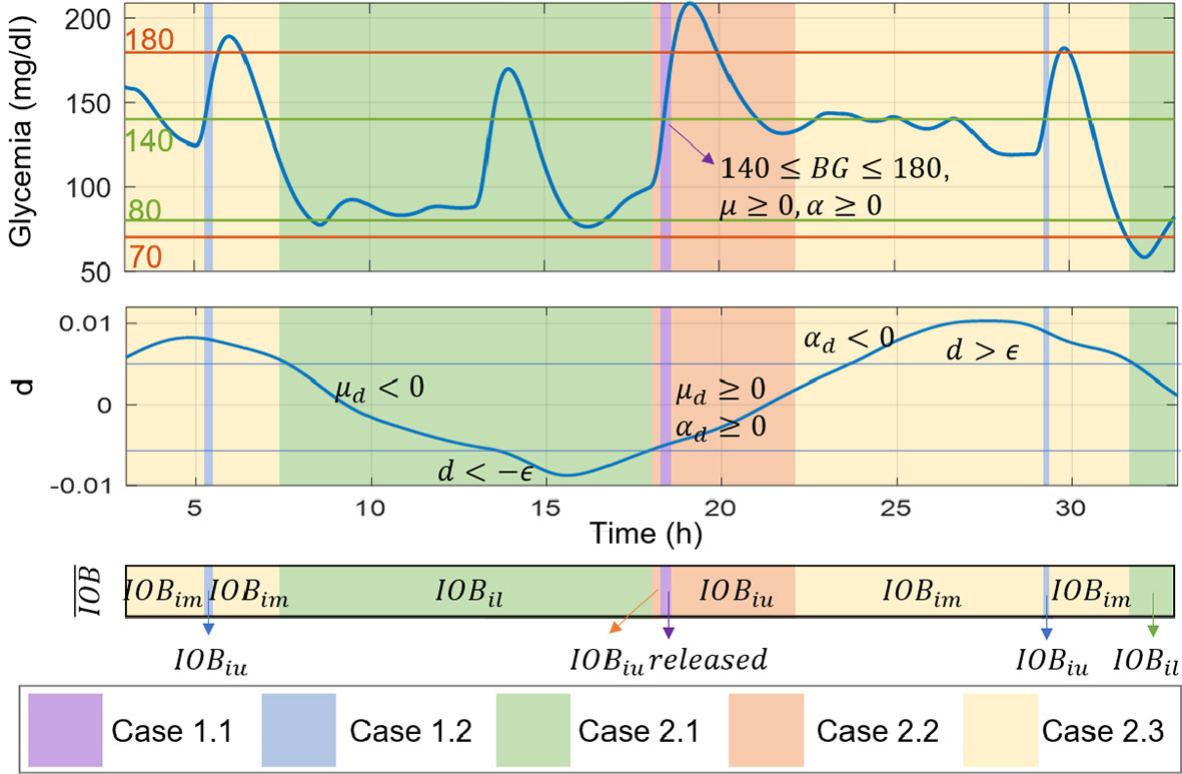
Illustrative scenario for the IOB constraint selection according to signals of glycemia, the estimated mismatch, and their first and second time derivatives.

A better visualization of the IOB constraint selection, using the cases described, can be seen in **Figure 3**. It is to clarify that the value of *ϵ* is set to consider the moments in which the physiological variations reach the higher andlower values. For the results here presented, a population value of *ϵ =* 0.005 was set from previous analysis on the behavior of *d* among the virtual subjects. Nevertheless, this parameter could be personalized by running the estimator in an open-loop treatment period to evaluate the magnitude of *d*. This implies that for the application of this control strategy, the collection of data prior to the activation of the control is a requirement. This practice is common in clinical studies, where data of each patient is collected prior to the clinical study to identify the prediction model, tune the estimator and controller matrices, and other parameters specific of the strategy (7).

After selecting the IOB constraint, the IOB of the subject is estimated using model (3) and a shorter sampling time Δ*t* than the one of the insulin pump *T*. At each time Δ*t*, the IOB is compared to the selected constraint, constructing the function $\sigma (\Delta t)=\bar{IOB}-IOB(\Delta t)$ . According to the sign of *σ*, the switching signal *ω* is determined: 


(8) $$\omega (\Delta t)=\{\begin{array}{cc} 1 & if\;\sigma (\Delta t)\geq 0\\ 0 & otherwise \end{array}.$$

Given the high frequency commutation between the values of *ω*, a chattering effect is obtained. To avoid it, the signal *ω* is smoothed by computed the gain *γ*(*k*) as the average of *ω*:


$$\gamma (k)=\frac{1}{n}\sum_{t=1}^{n}\omega (t),$$

With *n* = *T*/Δ*t* the number of samples of *w* obtained during the pump updating period *T*. Finally, this gain is the one used to modulate the control action of the iZMPC.

## 3 Results

To evaluate the control strategy, 10 virtual adult subjects were simulated by identifying the model parameters from the commercially available version of the UVA-Padova T1DM Simulator (36). A 36-hour scenario was selected, the sampling time *T* was set as 5 minutes, and the daily pattern of carbohydrate intake was 7:00h (55g), 10:00h (20g), 13:00h (90g), and 19:00h (70g). Intra-day variability was introduced in the simulation scenario by modifying three of the model parameters. This was done by considering circadian variability with 30% amplitude of the form:


$$p_{i}(t)=p_{i}^{*}\left(1+0.3\sin\left(\frac{2\pi }{1440}\right)t+2\pi RND\right),$$

where $p_{i}^{*}$ , with *i* = 0,1,3, is the nominal value of the parameters identified for each virtual subject and associated to the hepatic autoregulation, the insulin sensitivity, and the endogenous glucose production, and *RND* is a randomly uniformly generated number between 0 and 1 (38, 39). It should be noted that the parameter *p*_3_ is 180° out of phase with respect to *p*_0_ and *p*_1_ such that the three parameters induce hypoglycemia or hyperglycemia at the same time and thus prevent the effect of the variations of each parameter from compensating each other.

The iZMPC controller with interval safety layer (iZMPC-ISL) is compared with the iZMPC without the layer, and the iZMPC with a safety layer (iZMPC-SL) that uses the constraint $\bar{IOB}=IOB_{im}$ all the time. This with the purpose of putting in evidence the benefits of adapting the IOB constraint to avoid hypoglycemia and react against hyperglycemia episodes.

**Figure 4:**
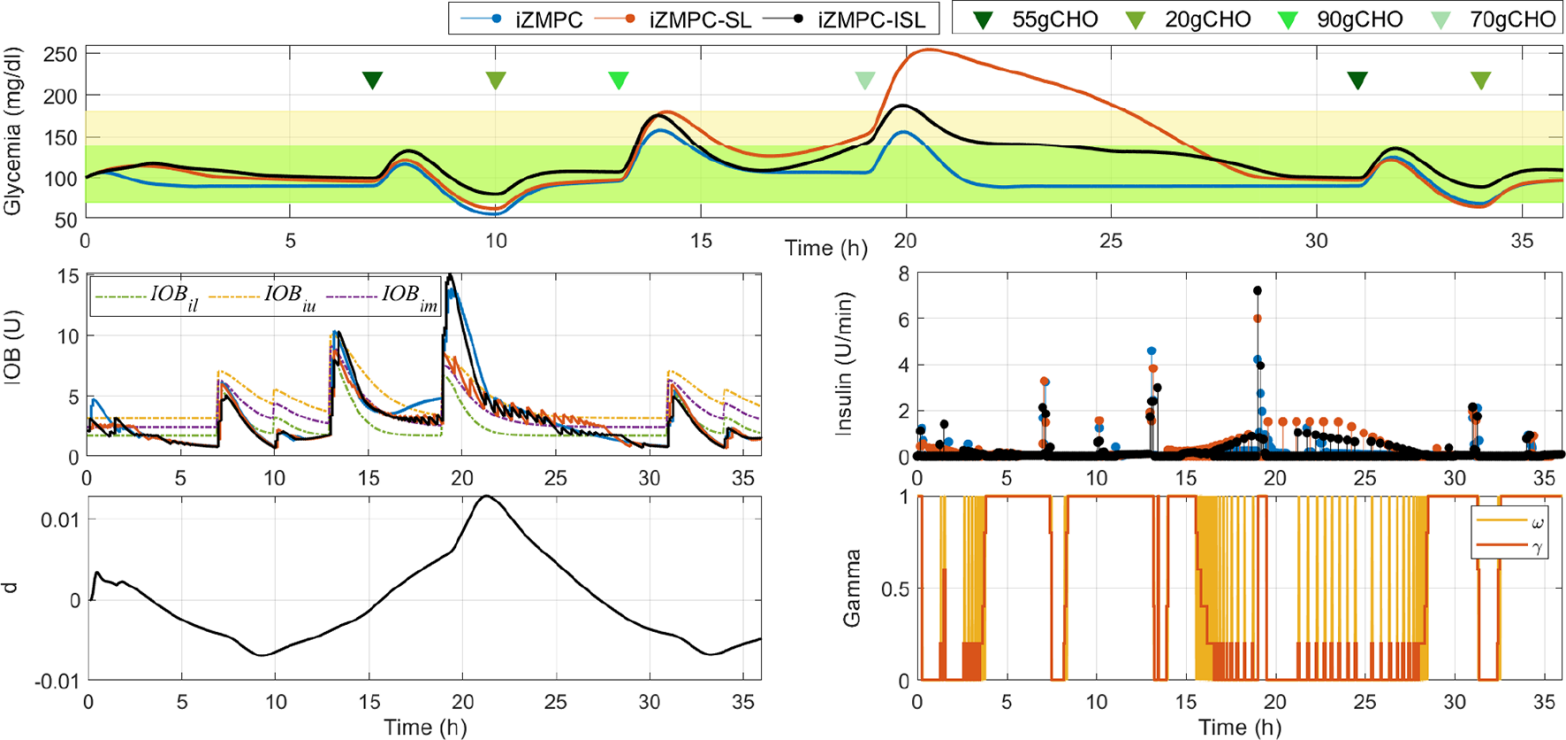
Comparison of the system evolution with the iZMPC and iZMPC-ISL.

In **Figure 4**, an illustrative example of virtual subject #10 is depicted. The Figure shows the glycemia, IOB, insulin, the estimated plant-model mismatch (*d*), and the gain *γ* that modulates the control action of the iZMPC. As shown, when using the iZMPC (blue lines) the system presents hypoglycemia episodes because of the delivery of insulin doses higher than the required for the situation of the subject (see that at 7:00h, the variations in the subject induce hypoglycemia). Next, the iZMPC-SL (red lines) is tested, but it can be seen the difficulties of setting one single constraint. If the constraint is too high to counteract variations inducing hypoglycemia, then the insulin doses are higher than the required, and if the constraint is lower than the required to compensate for variations inducing hyperglycemia (see the range between 15:00h and 25:00h) the controller is constrained and thus the insulin doses are not enough to low BG levels. To solve these issues, it can be seen that the iZMPC-ISL (black lines) uses the lower bound *IOB_il_* as IOB constraint when detecting variations towards hypoglycemia, changes to the middle or upper band when detecting variations towards hyperglycemia and release the constraint when the BG levels and its tendency allow to do it (see that the IOB constraint is exceeded at 19:00h). It is also noteworthy that the safety layer seems to almost override the controller during the night, as the control action generated by the iZMPC is highly attenuated. This shows that the safety layer can be decisive at the expense of the iZMPC. However, this situation is desired in diabetes treatment since the control action is precisely canceled when a risk of hypoglycemia is predicted, and therefore, insulin administration must be reduced or suspended. In addition, by using the interval model, the IOB boundary is established at the limit that is actually needed to compensate for current patient BG levels, trend, and detected physiological variations.

Next, by considering the same simulation scenario, the sensor noise was added to obtain the population outcomes. To that end, the CGM signal based on Dexcom G5 mobile devices was used. Its model and parameters can be seen in detail in (44). For each virtual subject, 10 simulations were performed, thus, 100 different cases were generated. The reported metrics of the population with each evaluated controller can be seen in **Table 3**. These consist of the mean BG (mg/dl), standard deviation (SD) of BG (mg/dl), coefficient of variation (CV) of BG (%), time percentage of BG in each range (%), and number of events in range. The outcome indexes are reported as mean ± SD for normally distributed data and as median (interquartile range) otherwise (45).

**Table 3:** Performance comparison of the iZMPC, iZMPC-SL, iZMPC-OLSL, and iZMPC-ISL.

Strategy	iZMPC	iZMPC-SL	iZMPC-OLSL	iZMPC-ISL
Mean BG (mg/dl)	99.4 ± 5.6	148.3 ± 18.9	112.2 ± 6.0	120.0 ± 7.6
SD (mg/dl)	29.8 ± 5.7	65.1 (21.0)	41.0 (14.2)	32.9 ± 7.1
CV (%)	30.0 ± 8.0	44.5 ± 8.3	37.5 ± 7.4	27.4 ± 5.6
Time percentage of BG (%)				
<54 mg/dl	2.1 ± 4.2	0 (2.8)	1.5 (5.9)	0 ± 0
<70 mg/dl	13.7 ± 6.5	5.8 (6.3)	9.9 (8.3)	0 ± 2.9
70–140 mg/dl	77.1 ± 7.4	48.6 ± 11.7	68.0 ± 9.5	76.3 ± 16.0
70–180 mg/dl	83.8 ± 6.7	61.9 (18.0)	82.8 ± 12.1	92.7 ± 5.8
>180 mg/dl	2.4 ± 3.7	32.5 (14.0)	7.1 (7.2)	6.3 ± 5.5
>250 mg/dl	0 ± 0	7.6 (11.9)	0 ± 1.8	0 ± 0
Number of events of BG				
<54 mg/dl	1 (2)	0 (1)	1 (1)	0 (0)
<70 mg/dl	3.5 (2)	1 (1)	2 (1)	0 (1)
>180 mg/dl	1 (2)	3 (3)	2 (1)	2 (1)
>250 mg/dl	0 (0)	1 (1)	0 (1)	0 (0)

**Figure 5:**
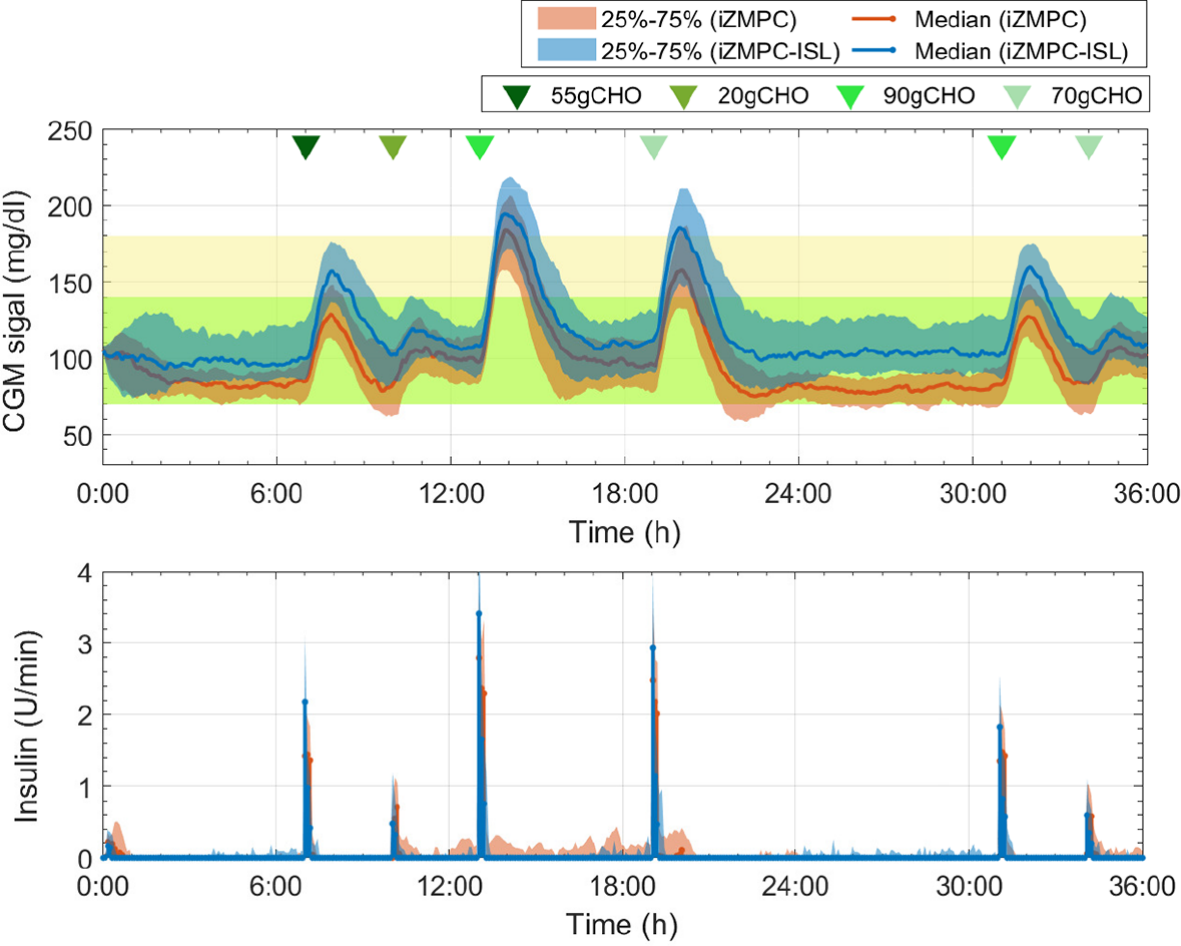
Comparison of the system evolution with the iZMPC and iZMPC-ISL.

**Figure 5** shows the comparison of the iZMPC and iZMPC-ISL. It is evident how the iZMPC-ISL significantly reduces hypoglycemia events without dangerously increasing BG levels. This is mainly achieved by reducing the insulin doses when required. The iZMPC-ISL *vs*. iZMPC improves the system performance in terms of time percentage in normoglycemia (92.7 ± 5.8 *vs*. 83.8 ± 6.7), reduces the time in hypoglycemia (0 ± 2.9 *vs*. 13.7 ± 6.5), the time in hyperglycemia increases (6.3 *pm* 5.5 *vs*. 2.4 ± 3.7) without obtaining events of severe hyperglycemia (BG > 250mg/dl). The complete outcomes can be seen in **Table 3**.

**Figure 6:**
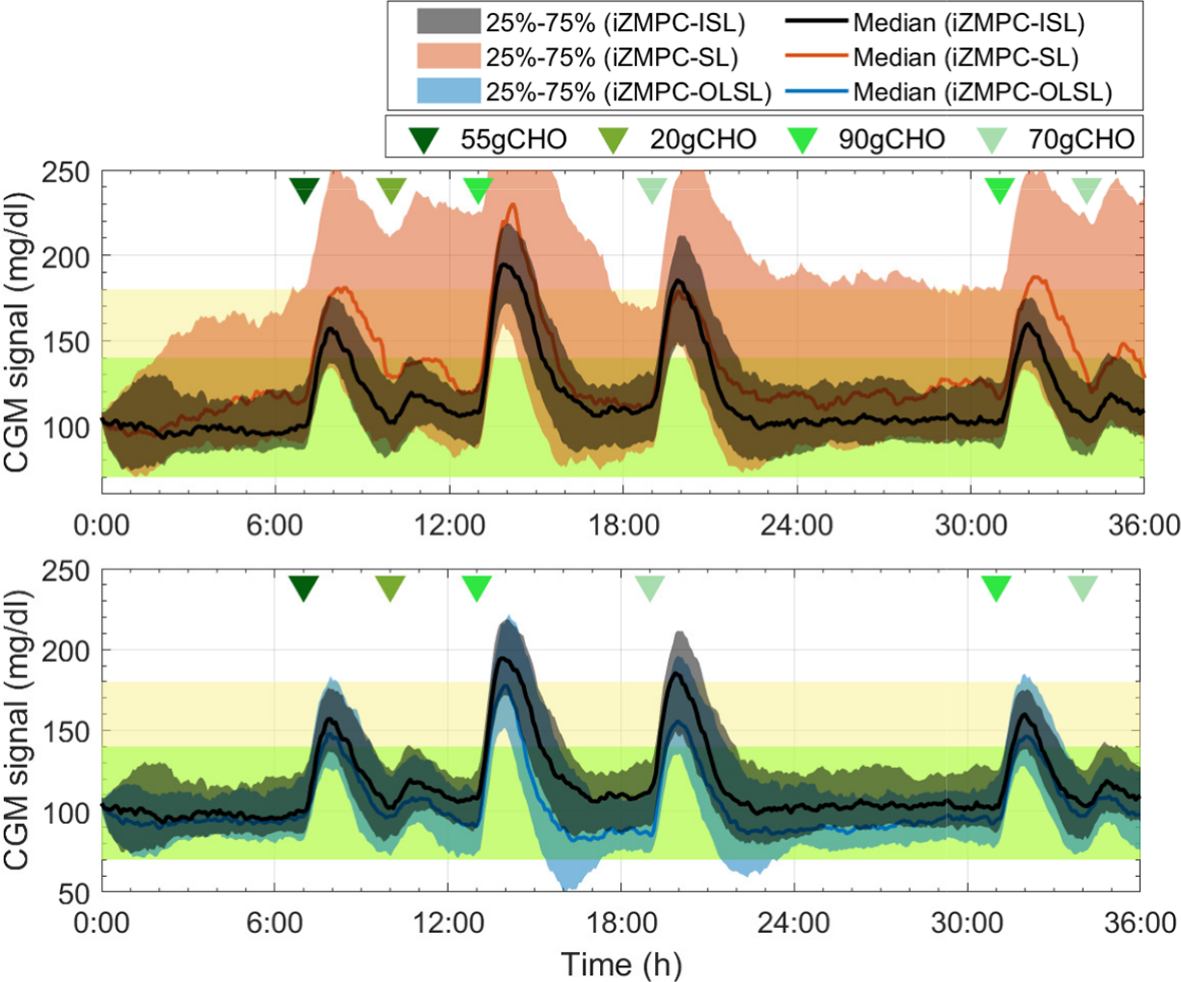
Comparison of the system evolution under the iZMPC-SL, iZMPC-OLSL, and iZMPC-ISL.

In addition, **Figure 6** shows the comparison of the iZMPC-ISL with two other strategies. First, the population results obtained with the iZMPC-SL are depicted. It can be seen how this strategy is harmful to compensate for hyperglycemia episodes. In fact, the time in hyperglycemia corresponds to 32.5% (see **Table 3**). As explained before, this behavior occurs because the IOB is constrained with the same boundary all the time, and thus, when the variations towards hyperglycemia occur(which have a time-random component), the control action is restricted, and the insulin doses delivered are not enough to counteract the variations.

Secondly, the strategy is compared with the one developed in (10), which uses an open-loop SL (here denoted as iZMPC-OLSL). This strategy was a first attempt of using information of the plant-model mismatch to change the IOB constraint. However, two limitations were detected: (i) the IOB constraint is computed as *β* times the IOB evolution considering the open-loop treatment, where the *β* factor is empirically established, and (ii) the rules for adapting the constraint dependedonly on the sign of *d*, and thus, there is not prediction regarding the changes in the variations (as in the iZMPC-ISL where the first and second time derivatives are used), leading to cases in which aggressive insulin doses are allowed precisely in the moments in which the variations start to decrease, which can cause hypoglycemia. As seen in **Figure 6**, the iZMPC-OLSL manages to avoid high BG levels and the hypoglycemia episodes in the first meals, nevertheless, for large meals late hypoglycemia occurs. Meanwhile, with the iZMPC-ISL, these events are avoided by using the first and second time derivatives of glycemia and the estimated mismatch to adapt the IOB constraint.

## 4 Conclusions

A control strategy for the artificial pancreas was introduced using an impulsive offset-free MPC coupled with an interval safety layer. The approach is intended to steer glycemia to the target zone while reducing the risk of hypoglycemia events. To that end, information of the parameter uncertainty is considered by means of an interval IOB model and the estimation of the plant-model mismatch with an augmented state estimator. The interval model establishes possible IOB boundaries to constraint the control action. While the current estimated mismatch, combined with the estimation of glycemia and their first and second time derivatives help to select the most suitable constraint for the current situation of the subject. In this regard, the controller changes between aggressive and conservative control actions to counteract hyperglycemia and hypoglycemia induced variations. This formulation achieved satisfactory results in an adult cohort and was compared with 3 different control approaches that vary the use of the safety layer. Future research includes the use of personalized information of the subject to establish some parameters, and/or the formulation of a function that explicitly relates the IOB constraint selection with the magnitude of the estimated variations.

## Data Availability Statement

The original contributions presented in the study are included in the article/supplementary material. Further inquiries can be directed to the corresponding author.

## Author Contributions

MV-T developed the code of the control strategy and performed the *in-silico* tests. MG-J contributed with the theoretical framework of the study about interval models. FL-V and PR conceived the study and analysis of the results, FL-V contributed with the theoretical framework of the safety layer, and PR with the framework of impulsive MPC. All authors contributed to draft the manuscript and approved the submitted version.

## Funding

This work was supported by Minciencias (Colombia) with Grant 110180763081.

## Conflict of Interest

The authors declare that the research was conducted in the absence of any commercial or financial relationships that could be construed as a potential conflict of interest.

## Publisher’s Note

All claims expressed in this article are solely those of the authors and do not necessarily represent those of their affiliated organizations, or those of the publisher, the editors and the reviewers. Any product that may be evaluated in this article, or claim that may be made by its manufacturer, is not guaranteed or endorsed by the publisher.

## Appendix A1

In this Appendix, the stability, observability, and controllability of the discretized model (1) are analyzed. Furthermore, an analysis on the stability of the interval model is presented.

### A1.1 Stability

The discretized system (1) maintains the stability of the original impulsive system (determined by the eigenvalues of *A_c_*), as the discretization is a mapping with the exponential function $\left(A=e^{A_{c}T}\right)$ . Since the eigenvalues of *A_c_* are real and negative, then the eigenvalues of the discrete system go to unit circle of the complex plane. Thus, maintaining the property of stability. It is noteworthy that this property does not depend on the value of *T*, as for all real and negative eigenvalues of *A_c_* the exponential function maps them between 0 and 1.

### A1.2 Observability

To implement the Kalman Filter, model (1) has to be observable. For impulsive systems, the observability criterion has been established in (46), where it is proved that a linear impulsive system is observable on some finite time interval if *Rank*[*C^T^ A^T^C^T^* …(*A^n-^* ^1^ )*^T^ C^T^*] = *n*.

The symbolic observability matrix for model (2) is:

$$\begin{array}{l} Obs\\ =\left[\begin{array}{lllll} 1 & -p_{o} & p_{0}^{2} & -p_{0}^{3} & p_{0}^{4}\\ 0 & -p_{1} & p_{o}p_{1}+\frac{p_{1}}{p_{4}} & -\frac{\left(p_{0}p_{1}+p_{1}/p_{4}\right)}{p_{4}}-p_{0}^{2}p_{1} & p_{0}^{2}(p_{0}p_{1}+\frac{p_{1}}{p_{4}})+\frac{\left(p_{0}p_{1}+p_{1}/p_{4}\right)}{p_{4}^{2}}\\ 0 & 0 & -\frac{p_{1}}{p_{4}} & \frac{\left(p_{0}p_{1}+p_{1}/p_{4}\right)}{p_{4}}+\frac{p_{1}}{p_{4}^{2}} & -2\frac{\left(p_{0}p_{1}+p_{1}/p_{4}\right)}{p_{4}^{2}}-\frac{p_{1}}{p_{4}^{3}}-\frac{\left(p_{0}^{2}p_{1}\right)}{p_{4}}\\ 0 & p_{2} & -p_{o}p_{2}-\frac{p_{2}}{p_{5}} & \frac{\left(p_{0}p_{2}+p_{2}/p_{5}\right)}{p_{5}}+p_{0}^{2}p_{2} & -p_{0}^{2}(p_{0}p_{2}+\frac{p_{2}}{p_{5}})-\frac{\left(p_{0}p_{2}+p_{2}/p_{5}\right)}{p_{5}^{2}}\\ 0 & 0 & \frac{p_{2}}{p_{5}} & -\frac{\left(p_{0}p_{2}+p_{2}/p_{5}\right)}{p_{5}}-\frac{p_{2}}{p_{5}^{2}} & (2(p_{0}p_{2}+\frac{p_{2}}{p_{5}}))/p_{5}^{2}+\frac{p_{2}}{p_{5}^{3}}+\frac{\left(p_{0}^{2}(p_{2}\right)}{p_{5}} \end{array}\right], \end{array}$$

with determinant given by $det=p_{1}^{2}p_{2}^{2}\frac{p_{4}^{4}+p_{5}^{4}-4p_{4}p_{5}^{3}-4p_{4}^{3}p_{5}+6p_{4}^{2}p_{5}^{2}}{p_{4}^{5}p_{5}^{5}}$ . The determinant satisfies *det ≠* 0 except for *p*_1_ = *p*_2_ =0, which never occurs due to the physiological meaning of the parameters.

### A1.3 Controllability

To implement the MPC, model (1) has to be controllable. The controllability criterion for impulsive control systems has been discussed in (1), where it is established that a system is impulsively controllable if and only if *rank*[*B AB A^2^B* …*A^n-^* ^1^B] = *n*. For the diabetes application, it is to clarify that, the state variables *x*_4_. *x*_5_ only depend on the meal disturbance *r*, and thus, they are not controllable (they are not affected by insulin). Therefore, the controllability of the model is evaluated without considering these states. The symbolic controllability matrix of the model is:

$$Co=\left[\begin{array}{ccc} 0 & 0 & -p_{2}/p_{4}^{2}\\ 0 & 1/p_{4}^{2} & -2/p_{4}^{3}\\ 1/p_{4} & -1/p_{4}^{2} & 1/p_{4}^{3} \end{array}\right],$$

which has full rank for all values of the parameters except for *p*_4_ = 0 or *p*_2_ = 0. However, as *p*_4_ represents the time-to-maximum of effective insulin concentration, and *p*_2_ is the carbohydrate bio-availability, their values are never zero.

### A1.4 Stability of the Interval Model

The model in (5) can be rewritten in the form *x* (*k* + 1) = *Ax*(*k*) + *Bu*(*k*) when considering a system of 6 state variables ([*x*_3_ *_low_ x*_3_ *_upper_ x*_23_ *_low_ x*_23_ *_upper_ x*_2_ *_low_ x*_2_ *_upper_*]). The resulting matrix *A* of the interval systemis:

$$A=\left[\begin{array}{cccccc} 1-\frac{1}{p_{1}} & 0 & 0 & 0 & 0 & 0\\ \frac{1}{p_{2}} & 1-\frac{1}{p_{1}} & 0 & 0 & 0 & 0\\ 0 & 1 & 0 & -1 & 0 & 0\\ 0 & 0 & 0 & 1-\frac{1}{p_{2}} & 0 & 0\\ 0 & 0 & 0 & \frac{1}{p_{1}} & 1-\frac{1}{p_{2}} & 0\\ -1 & 0 & 0 & 0 & 1 & 0 \end{array}\right]$$

with eigenvalues [0 0 (*p*1–1)/*p*1 (*p*1–1)/p1 (*p*2–1)/*p*2 (*p*2–1)/p2]*'*. These eigenvalues are always within the unit circle of the complex plane. Therefore, the interval model is asymptotically stable.
